# The Roles of Dietary Bioactive Compounds in Alleviating Inflammatory Bowel Disease‐Associated Colorectal Cancer

**DOI:** 10.1002/fsn3.70432

**Published:** 2025-06-20

**Authors:** Jin‐Wei Zhao, Wei‐Yi Zhao, Zhong‐Yang Yu

**Affiliations:** ^1^ Department of Hepatopancreatobiliary Surgery of Second Hospital of Jilin University Jilin University Changchun China; ^2^ YanBian Hospital Yanbian University Yanji Jilin Province China; ^3^ State Key Laboratory of Cardiovascular Diseases, Shanghai East Hospital, School of Medicine Tongji University Shanghai China

**Keywords:** food‐derived bioactive ingredients, inflammatory bowel disease (IBD)‐associated colorectal cancer (CRC), mechanism, treatment and prevention effects

## Abstract

Inflammatory bowel disease (IBD)‐associated colorectal cancer (CRC) is a serious global health issue. Owing to the successful mimicking of the entire process of CRC induced by IBD, azoxymethane (AOM) and dextran sulfate sodium (DSS)‐induced colitis‐associated cancer (CAC) animal models have been widely used to study the carcinogenic mechanisms of IBD‐associated CRC and aid in the discovery of new candidate target drugs for CRC treatment. This article summarizes the molecular characteristics and mechanisms of the AOM/DSS‐induced CAC animal model and reports the effects and mechanisms of dietary active ingredients such as phenolics, flavonoids, terpenes, polysaccharides, alkaloids, and other food extracts in the prevention and treatment of AOM/DSS‐induced CAC in preclinical trials. Further research and application of dietary active ingredients against AOM/DSS‐induced CAC are needed in the future. In conclusion, this study may provide insights into the potential application of active dietary ingredients in the treatment of CAC.

## Graphical abstract

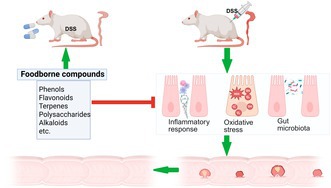

**Abbreviations**
6‐G: 6‐gingerol (6‐G)
ACF: aberrant crypt foci
AFE: flavonoid‐rich extracts from okra flowers
AOM: azoxymethane
AP: apple polysaccharide
ARE: antioxidant response elements
BL: barley leaf
BPIS: bound polyphenol of the inner shell
CAC: colitis‐associated cancer
CARV: carvacrol
Cdk4: cyclin‐dependent kinase 4
CRC: colorectal cancer
CS: carnosol
CUR: curcumin
DALMs: dysplasia‐associated lesions or masses
DMD: 1,2‐dimethylhydrazine
DRB: defatted rice bran
DSS: dextran sulfate sodium
EMR1: epidermal growth factor‐like domain‐containing mucin‐like hormone receptor 1
ETBF: enterotoxigenic *Bacteroides fragilis*

FMT: fecal microbiota transplantation
FS: fisetin
gal‐3: galactoside‐binding lectin
GEN: genistein
GLN: glutamine
GPx: glutathione peroxidase
GTN: goniothalamin
HDC: histidine decarboxylase
HO‐1: heme oxygenase‐1
IBD: inflammatory bowel disease
IECs: intestinal epithelial cells
IL‐6: interleukin‐6
Kae: Kaempferol
mCur: micelle‐formulated curcumin
MDSCs: myeloid‐derived suppressor cells
MPs: mononuclear phagocytes
MYR: myricetin
OLG: Oligonol
PB2: procyanidin B2
PC: phosphatidylcholine
PC: phycocyanin
PE: phosphatidylethanolamine
PL: procyanidin B2
PT: parthenolide
RA: rosmarinic acid
RE: resveratrol
ROS: reactive oxygen species
S1P: sphingosine‐1‐phosphate
SeN: selenoneine (SeN)
TRI: tricin
Vit: vitexin
WPE: walnut phenolic extract
XO: xanthine oxidase

## 1 Introduction

Colorectal cancer (CRC) is the third most commonly diagnosed cancer and the second leading cause of cancer‐related deaths worldwide. It is a major global health concern (Sung et al. 2021; Papamichael et al. 2015). According to data from GLOBOCAN, there were approximately 19.3 million new cases of cancer and 10 million cancer deaths globally in 2020 (Sung et al. 2021). Over the last 2 years, the incidence of CRC in men has risen from 5% to about 10%. However, with the notable rise in cases among the elderly, the global incidence of CRC is projected to more than double by 2035, especially in less developed countries (Papamichael et al. 2015).

In the progression of inflammation‐dysplasia‐carcinogenesis in colitis‐associated CRC (CAC), inflammation is key to the development of CAC (Kameyama et al. 2018; Porter et al. 2021). Chronic inflammation plays a role in the development and progression of CRC. Inflammatory bowel diseases (IBDs), such as Crohn's disease and ulcerative colitis are known to significantly elevate the risk of CRC (Lakatos and Lakatos 2008; Lutgens et al. 2013), indicating that long‐term inflammation triggers genetic alterations that raise the likelihood of developing CRC. For instance, mutations that deactivate the adenomatous polyposis coli (APC) gene typically arise in advanced stages of CRC formation (Cooper et al. 2001).

**FIGURE 1:**
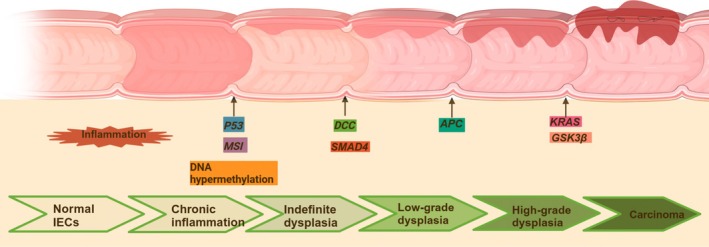
Inflammatory bowel disease (IBD) as a high‐risk factor for colorectal cancer.

Reports indicate that microsatellite instability and DNA hypermethylation arise early in cancer progression (Fleisher et al. 2000). Loss‐of‐function mutations of p53 can also be detected early in CAC (Cooks et al. 2013). Besides APC mutations, KRAS mutations usually occur in the later stages of CAC development, like high‐grade dysplasia (Lang et al. 1999). Earlier research has indicated that alterations in the deleted colon cancer gene (DCC) and SMAD4 on chromosome 18q take place during the progression from indeterminate atypical hyperplasia to low‐grade dysplasia (Tarafa et al. 2000). Changes in APC and modifications to the glycogen synthase kinase‐3β (GSK3β) gene are also regarded as crucial factors for the progression from high‐grade dysplasia to carcinoma (Itzkowitz 2006) (Figure 1).

Although tremendous efforts have been made to develop effective therapies for CRC, advanced‐stage cancer remains difficult to treat and has low survival rates (DeSantis et al. 2014). Therefore, There is a pressing need to create effective and relatively safe agents to slow the progression of CRC by reducing chronic intestinal inflammation. Developing new drugs and treatments for CRC requires preclinical testing in experimental models. The azoxymethane (AOM) and dextran sulfate sodium (DSS)‐induced colitis‐associated cancer (CAC) animal model is widely used because it successfully mimics the full process of IBD‐associated CRC (IBD‐CRC). This model is essential for studying the mechanisms of IBD‐CRC carcinogenesis, understanding the pathological changes and principles of CRC development, and identifying potential new drugs for CRC treatment (Dzhalilova et al. 2023). The AOM/DSS experimental animal model mimics the ongoing damage and repair process in the intestinal lining through the combined effects of the chemical carcinogen AOM and the inflammatory agent DSS. Carcinogenesis is first triggered by AOM, and then the experimental mice are continuously exposed to DSS, creating a model of cancer development alongside ulcerative colitis in the short term (Dzhalilova et al. 2023).

Interestingly, medical scientists are working to develop new therapeutic strategies that may include natural products from food sources, as growing evidence from both laboratory and live animal studies shows that compounds derived from diet can effectively treat IBD and related cancers through various mechanisms (Zhao and Jiang 2021).

**FIGURE 2:**
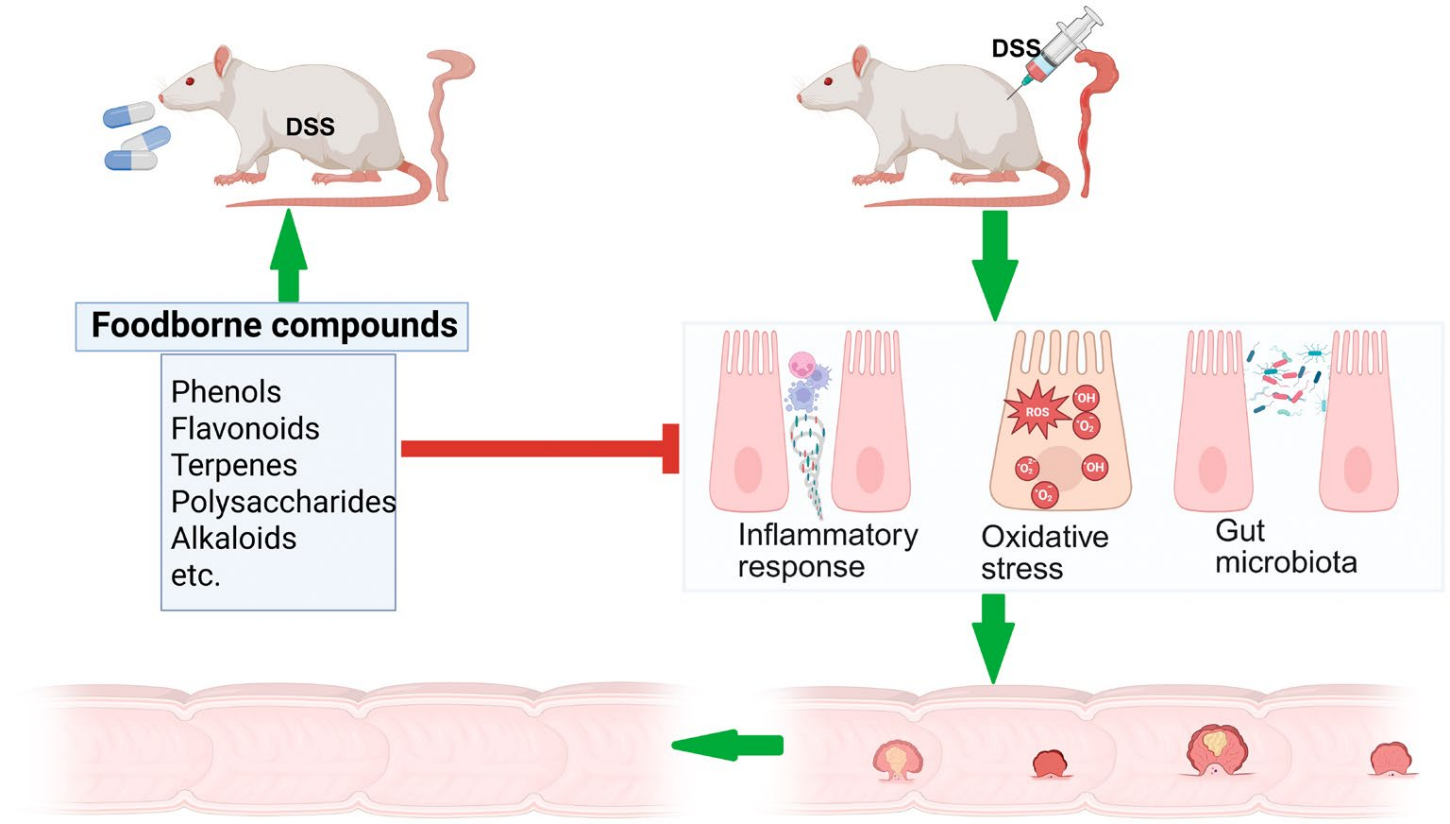
This article summarizes the molecular characteristics and mechanisms of the AOM/DSS‐induced CAC animal model, and reports on the preventive and therapeutic effects and mechanisms of phenolic compounds, flavonoids, terpenoids, polysaccharides, basic alkaloids, and other food extracts in preclinical trials for AOM/DSS‐induced CAC. Further research is still needed to explore and apply dietary active ingredients for the prevention and treatment of AOM/DSS‐induced CAC. This study may provide insights into the potential application of active dietary ingredients in CAC treatment.

In this review, we provide information on the molecular characteristics and pathogenesis of AOM/DSS‐induced CAC models, as well as the therapeutic effects and mechanisms of dietary compounds on AOM/DSS‐induced CAC (Graphic abstract as illustrated in Figure 2). Studying these innovative products in animal models may inspire the implementation of clinical trials and promote their application in medical practice.

## 2 Molecular Characteristics in AOM/DSS‐Induced CAC

**FIGURE 3:**
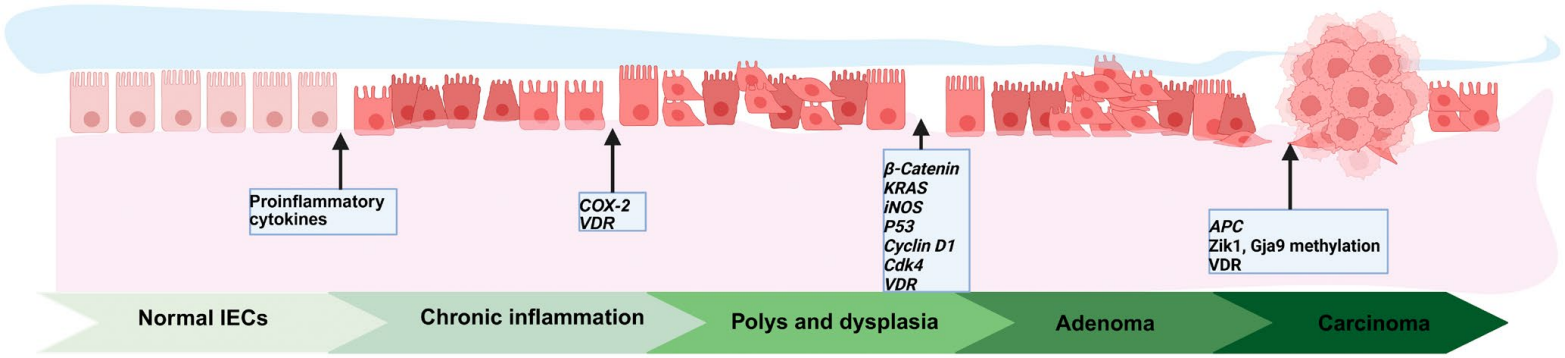
Chronic inflammation leads to genetic mutations in AOM/DSS‐induced CAC. Intestinal inflammation is a hallmark of CAC, and in the inflammation‐dysplasia‐carcinoma sequence of CAC, mutations in *KARS, P53* occur in the early phase of the development of CAC, whereas the loss of APC, Zik1 and Gja9 DNA hypomethylation occurs late in the development of CAC. On the other hand, mutations in β‐catenin, iNOS, COX‐2, Ckd4, and Cyclin D1 are characteristic early phenomena in CAC. The map was created using biorender.

CAC originates and develops in the chronic inflammatory mucosa, progressing through stages of uncertain hyperplasia. These stages typically include flat dysplasia, low‐grade dysplasia, high‐grade dysplasia, adenoma, and eventually cancer (Kameyama et al. 2018; Porter et al. 2021). In animal models of CAC induced by AOM/DSS, inflammation can induce the development and progression of colon tumors, suggesting that chronic inflammation can lead to genomic changes that increase the risk of CRC (Ullman and Itzkowitz 2011) (as shown in Figure 3). Maltzman et al. (1997) used antitumor suppressor protein APC antibodies to demonstrate for the first time that wild‐type APC protein is absent in AOM‐induced colon tumors in mice. Studies have shown notable β‐catenin staining in both the cytoplasm and nuclei of all colon adenomas and cancer tissues. Immunoblot analysis indicated an elevated level of free β‐catenin in tumor cells (Takahashi et al. 1998), Additionally, further investigation revealed a high occurrence of mutations in the β‐catenin gene across all the colon tumors studied (Takahashi, Nakatsugi, et al. 2000). Takahashi M and colleagues also studied how β‐catenin and K‐ras gene mutations relate to the expression of iNOS and COX‐2 induced by AOM during the development of colon cancer in rats. Their findings showed that β‐catenin gene mutations were present in varying amounts across dysplastic aberrant crypt foci (ACF), adenomas, and adenocarcinomas, with some cases of K‐ras mutations also detected in hyperplastic ACF and adenocarcinomas. iNOS expression was seen in almost all damaged areas where β‐catenin changes occurred. COX‐2 expression was found in normal colonic epithelial cells and increased in adenomas and adenocarcinomas, with positive reactions limited to the epithelial cells of advanced adenocarcinomas (Takahashi, Mutoh, et al. 2000). In AOM/DSS‐induced CAC models, the movement of β‐catenin between the nucleus and cytoplasm occurs early in dysplasia‐associated lesions or masses, but not in flat dysplasia or cancer. Additionally, p53 plays little to no role in dysplasia or cance (Cooper et al. 2000). However, the role of the P53 gene in the formation of colon tumors induced by AOM is still not fully understood. Chang et al. found that the loss of p53 increases the development of CAC, especially flat lesions. As seen in humans, p53 acts as a protective factor in CAC in the DSS model. (Chang et al. 2007). A previous study examined the occurrence of K‐ras gene mutations. The findings showed that no mutations were present in normal mucosa, but K‐ras mutations were detected in ACF, adenomas, and adenocarcinomas in AOM/DSS‐induced CAC models (Vivona et al. 1993). In contrast, K‐ras mutations are rare in AOM‐induced mouse colon cancer (Takahashi, Nakatsugi, et al. 2000). Additionally, K‐ras mutations have not been found in colon tumors caused by 1,2‐dimethylhydrazine. These results suggest that activating the K‐ras gene is not necessary for colon cancer development (Jackson et al. 1999).

Moreover, in mice treated with AOM, there was a marked increase in the number of cells showing immunoreactivity for cyclin D1 and cyclin‐dependent kinase 4 (Cdk4) in early lesions and adenomas. In addition, the crypts displayed positive immunoreactivity for cyclin D1, indicating that the overexpression of cyclin D1 and Cdk4 happens early in the development of CAC (Wang et al. 1998).

Interestingly, recently, Chen et al. used bioinformatics methods to discover that the Vitamin D receptor (VDR) expression in the intestinal tissues of patients or mice with chronic intestinal inflammatory bowel cancer (CAC) and CRC was downregulated. Further modeling research on the progression from chronic intestinal inflammation to carcinogenesis revealed that, compared to the normal group, the expression of VDR protein in the model group was significantly reduced. Tumor formation was significantly increased in myeloid VDR knockout mice. These results strongly suggest that VDR plays an important role in the carcinogenesis induced by chronic intestinal inflammation (Chen et al. 2024).

DNA hypomethylation plays a causal role in tumorigenesis, possibly by promoting chromosomal instability (Gaudet et al. 2003). Similar to human CRC, in the AOM‐induced rodent CRC model, tumors exhibit global DNA hypomethylation, and Zik1 and Gja9 show cancer‐specific abnormal DNA methylation (Borinstein et al. 2010).

## 3 Molecular Mechanisms of AOM/DSS‐Induced CAC

**FIGURE 4:**
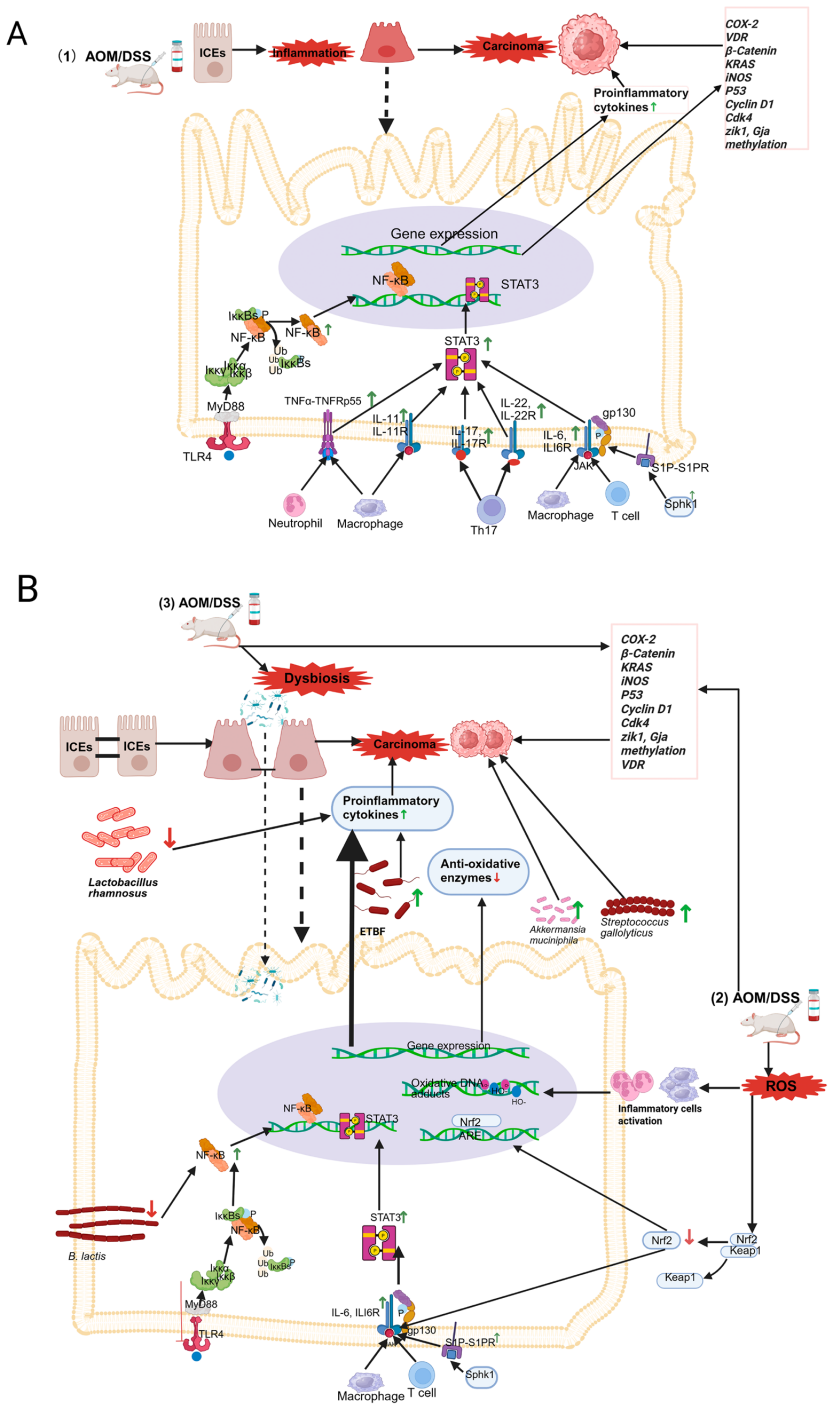
Molecular mechanisms of AOM/DSS‐induced CAC. (1) Choric inflammation. (2) Oxidative stress. (3) Dysbiosis. The map was created using biorender.

Mounting evidence shows that chronic inflammation is a risk factor for cancer. Chronic inflammation enhances CAC development through various mechanisms, including chronic inflammation, oxidative stress and gut dysbiosis, which induce DNA damage, activate oncogenes, and inactivate tumor suppressor genes to initiate colon tumorigenesis (as shown in Figure 4A,B).

### 3.1 The Role of Inflammation in AOM/DSS‐Induced CAC

The transcription factor NF‐κB regulates the integrity of the epithelial layer and the interaction between the mucosal immune system and the gut microbiome. Issues with NF‐κB signaling are central to the breakdown of intestinal immune balance, resulting in symptoms that resemble IBD (Nenci et al. 2007). Research has demonstrated that in AOM/DSS‐induced CAC models, deleting IKKβ in colonic epithelial cells significantly lowers tumor incidence, whereas deleting IKKβ in bone marrow cells results in a notable reduction in tumor size. These findings indicate that selectively inactivating the IKK/NF‐κB pathway in these two distinct cell types can reduce the link between inflammation and tumor development. Potential mechanisms include not only inhibiting apoptosis in advanced‐stage tumors but also connecting inflammation to cancer through IKKβ (Greten et al. 2004).

Research has demonstrated that interleukin‐6 (IL‐6) is a versatile cytokine regulated by NF‐κB that affects both epithelial and immune cells. IL‐6 plays a key role in promoting tumors during the early stages of CRC (CAC) development. In a similar vein, mice that lack IL‐6 in bone marrow‐derived cells or are IL‐6‐deficient show fewer tumors and a lower tumor burden. Further investigations reveal that IL‐6 produced by bone marrow‐derived cells not only boosts the growth of tumor‐initiating cells but also helps protect normal and precancerous intestinal epithelial cells (IECs) from cell death. The effects of IL‐6 on cell growth and survival are mainly driven by STAT3. The NF‐κB–IL‐6–STAT3 pathway is a crucial factor in regulating the growth and survival of tumor‐initiating IECs (Grivennikov et al. 2009). Sphingosine‐1‐phosphate (S1P) is crucial for the production of IL‐6, sustained activation of STAT3, and upregulation of the S1P receptor S1PR1. The SphK1/S1P/S1PR1 axis, located between NF‐κB and STAT3, is a key link between chronic inflammation and CAC (Liang et al. 2013).

A study by Popivanova et al. showed that in wild‐type mice, there is an increased expression of TNF‐α and infiltration of inflammatory leukocytes that express its primary receptor, p55 (TNF‐Rp55), in the lamina propria and submucosa of the colon. This leads to the development of multiple colon tumors. However, mice without TNF‐Rp55 show less mucosal damage, a reduction in macrophage and neutrophil infiltration, and reduced tumor formation, supporting the idea that TNF‐α plays a key role in the development and progression of CAC (Popivanova et al. 2008).

Research by Wang et al. showed that either removing neutrophils or blocking IL‐1β activity significantly lowers mucosal damage and tumor development in a mouse model of CAC induced by AOM/DSS. The pro‐cancer role of IL‐1β is mainly due to its promotion of IL‐6 secretion by intestinal resident mononuclear phagocytes (Wang et al. 2014). Additionally, IL‐11, which is triggered by colitis, activates STAT3 in the epithelial cells of colonic crypts. The activation and phosphorylation of STAT3 (pSTAT3) stay elevated from the hyperplastic dysplasia stage through to the tumor stages. Blocking IL‐11 signaling reduces the cancer development caused by colitis in AOM/DSS‐induced CAC models (Liang et al. 2013). Furthermore, Hyun et al. reported that both the number and average size of tumors significantly decrease in IL‐17A‐deficient mice, suggesting that depletion of IL‐17A can significantly reduce AOM/DSS‐induced CAC formation (Hyun et al. 2012). Interestingly, the administration of the small‐molecule drug Androvia (Andro) notably slows the progression of colitis and reduces tumor burden by inhibiting the NLRP3 inflammasome. Additional mechanistic studies showed that Andro induces mitophagy in macrophages, which restores mitochondrial membrane potential and leads to the inactivation of the NLRP3 inflammasome in AOM/DSS‐induced CRC models (Guo et al. 2014). It is worth noting that the study by Chen et al. suggests that in mid‐stage CAC mice, the expression of VDR protein is significantly reduced, and the absence of VDR protein seems to accelerate the progression of CAC, possibly due to changes in macrophage polarization. Vitexin has been shown to directly target VDR, binding to the VDR‐LBD domain, thereby promoting its translocation to the cell nucleus. This process then regulates the transcription of PBLD and affects macrophage polarization. These results indicate that the direct targeting of VDR by vitexin alters macrophage polarization and thus inhibits the progression of colitis to CRC (Chen et al. 2024).

### 3.2 The Role of Oxidative Stress in AOM/DSS‐Induced CAC

Increasing evidence suggests that inflammation‐driven oxidative stress plays a key role in causing mutations that lead to CAC. When inflammatory cells are activated, there is a rise in reactive oxygen species (ROS) at the site of inflammation. Chronic inflammation resulting in high levels of ROS can cause oxidative DNA damage, leading to mutations and disrupted cell growth regulation, which in turn may promote cancer development (Sawa and Ohshima 2006). Under oxidative stress, Nrf2 detaches from Keap1, translocates into the nucleus, and interacts with other transcription factors to activate genes with antioxidant response elements (Kobayashi and Yamamoto 2005; Motohashi et al. 2004). This results in the increased expression of various antioxidant enzymes, which helps decrease sensitivity to oxidative damage and cytotoxicity, whereas also reducing inflammation‐related damage and neutralizing ROS involved in inflammatory signaling pathways (Motohashi et al. 2004; Osburn et al. 2007; Zhu et al. 2006). Osburn et al. showed that disrupting the Nrf2 gene in mice greatly increases the development of colonic adenomas. On the other hand, an active Nrf2 signaling pathway helps trigger adaptive responses that counteract the cancer‐promoting effects of DSS (Osburn et al. 2007). Furthermore, in the AOM‐DSS model, mice without glutathione peroxidase (GPx) 3 tend to develop more tumors, and these tumors are of a higher grade of dysplasia (Barrett et al. 2013). In addition, a cocoa‐rich diet significantly decreases the development of crypts and buds and reduces the tumor size in mice treated with AOM/DSS, and mechanistic studies have revealed that cocoa acts as a potent inducer of Nrf2 and its downstream target genes, thereby inhibiting the occurrence and development of CAC. Additionally, a diet high in cocoa significantly reduces the development of crypts and buds and lowers tumor size in mice treated with AOM/DSS. Mechanistic studies have shown that cocoa is a powerful activator of Nrf2 and its target genes, which helps prevent the occurrence and progression of CAC (Pandurangan et al. 2015). Moreover, flavonoid‐rich extracts from okra flowers (AFE) significantly inhibit colitis and the formation of AOM/DSS‐induced CAC by influencing the Nrf2/IL‐6 signaling pathway (Deng et al. 2023).

### 3.3 The Role of the Gut Microbiota in AOM/DSS‐Induced CAC

Growing evidence suggests that the gut microbiota is essential in tumor development in the AOM/DSS‐induced CAC model. Giving vancomycin to AOM/DSS‐treated mice can reduce tumor growth by lowering the number of DNA damage‐triggered neutrophils in IECs (Tanaka et al. 2016) Additionally, enterotoxigenic *Bacteroides fragilis* (ETBF) is known to disrupt the intestinal barrier. When ETBF colonizes the gut, it causes an increase in pro‐inflammatory cytokines, which can trigger polyp formation (Hwang et al. 2020). Recently, the formation of bacterial biofilms has been considered a driving factor in the early development of CRC (Li et al. 2019; Chew et al. 2020). Notably, the buildup and colonization of *Streptococcus gallolyticus* in colon cancer tissues in AOM/DSS‐treated mice accelerate CRC progression (Zhang, Weng, et al. 2018). Furthermore, administering the mucin‐degrading bacterium *Akkermansia muciniphila* promotes IEC proliferation and increases susceptibility to CRC in mice (Wang et al. 2022).

Several studies have also shown that correcting the imbalance in the gut microbiota can reduce the onset and progression of CAC, offering evidence that such an imbalance contributes to the development of CRC. In AOM/DSS‐induced CAC, fecal microbiota transplantation (FMT) from healthy control mice effectively restores the disrupted gut microbiota and prevents tumor formation in CRC (Wang et al. 2019). Research using a CAC model revealed that *B. lactis* inhibits NF‐κB activation in IECs and suppresses acute colitis and CAC development (Kim et al. 2010). Another study indicated that the administration of *Bifidobacterium bifdum* mitigates intestinal tumor occurrence by modulating the gut microbiota (e.g., the enrichment of Ruminococcaceae and Lachnospiraceae, known as anti‐inflammatory factors) and metabolic composition, such as fatty acid metabolism pathwaysAnother study found that taking *Bifidobacterium bifidum* helps reduce the occurrence of intestinal tumors by influencing the gut microbiota—specifically increasing the presence of Ruminococcaceae and Lachnospiraceae, which are known to have anti‐inflammatory effects and by affecting metabolic processes like fatty acid metabolism pathways (Wang et al. 2020). Moreover, *Lactobacillus rhamnosus* treatment has been shown to slow tumor growth and decrease intestinal inflammation in AOM/DSS‐induced CAC by regulating inflammatory responses and lowering proinflammatory cytokines in the tumors (Silveira et al. 2020). *Lactobacillus reuteri* has a distinctive ability to convert histidine into histamine. When histamine‐producing *L. reuteri* is administered, it reduces both the number and size of tumors in AOM/DSS‐induced histidine decarboxylase (HDC)‐deficient CAC mice. In contrast, *L. reuteri* strains that lack histamine (HDC mutant strains) do not have the same effect on CAC suppression (Yang et al. 2011; Gao et al. 2017). In summary, the gut microbiota is likely to play a pathogenic role in CAC.

## 4 The Role of Food‐Derived Compounds in AOM/DSS‐Induced CAC

**FIGURE 5:**
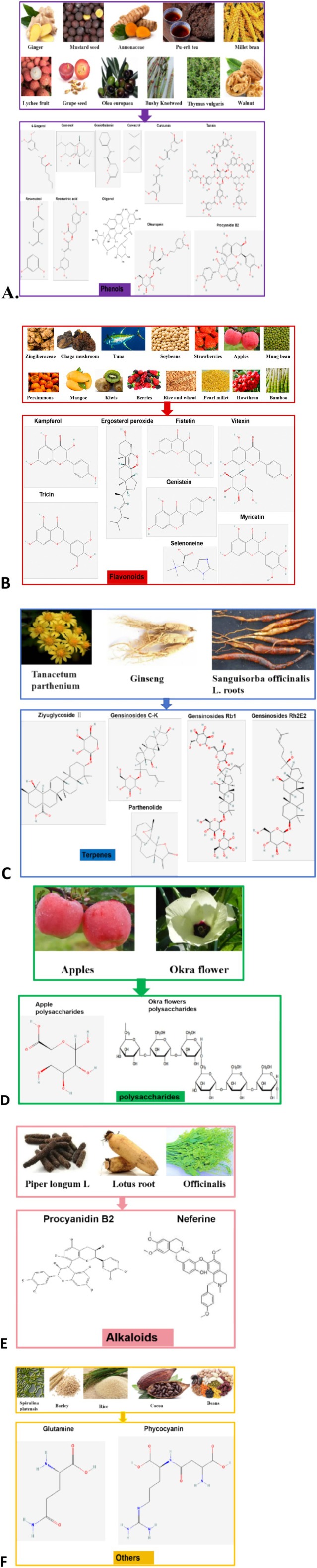
Foods and food‐derived bioactive compounds with effect on AOM/DSS‐induced CAC. (A) Foods and food‐derived phenols (purple color); (B) Foods and food‐derived flavonoids (red color); (C) Foods and food‐derived terpenes (blue color); (D) Foods and food‐derived polysaccharides (green color); (E) Foods and food‐derived active alkaloids (pink color); (F) Others (yellow color). The color map of molecular structures are sourced from PubChem, whereas black‐and‐white mapp of molecular structures are created using Biorender.

Many food‐derived products have been widely reported to prevent and treat AOM/DSS‐induced CAC. In vivo animal model studies have demonstrated that a single component of food‐derived products, especially phenols, flavonoids, terpenes, polysaccharides, and alkaloids, may be beneficial for the treatment of AOM/DSS‐induced CAC (Table 1 and Figure 5A–E).

**TABLE 1:** The role and mechanisms of food‐derived compounds in AOM/DSS‐induced CAC.

Type	Compounds	Diets sources	Does (time)	Molechular mechanisms	Authors (Ref.)
Phenols	6‐Gingerol	Ginger	50 mg/kg, twice week for 32 weeks	β‐catenin and cyclin D1↓; anti‐inflammation and antioxidant stress	Farombi et al. (2020)
	Canolol	Mustard seed oil, mustard seed oil	0.1%–0.3% of diets for 7 days	COX‐2, iNOS and TNF‐α↓; HO‐1↓; anti‐inflammation and antioxidant stress	Fang et al. (2013)
	Goniothalamin	Genus *Goniothalamus* (Annonaceae)	30 mg/kg, twice week for 3 weeks	Tissue IL‐6, IL‐17 and TNF‐α↓; stromal immune cell activation and NF‐κB↓	Vendramini‐Costa et al. (2017)
	Carvacrol	*Oreganum vulgare* essential oil	50 mg/kg, for 13 weeks	Anti‐inflammation and antioxidant stress	Arigesavan and Sudhandiran (2015)
	Curcumin	*Curcuma longa* Linn.	18 mg/day for 2 weeks; 1000 ppm and 5000 ppm for 16 weeks; 2% of diets for13 weeks; 500 mg/kg/day for 10 weeks	DNA CpG methylation of inflammation‐related genes; Axin2↓; Wnt/β‐catenin protein signaling pathway↓	Villegas et al. (2011); Murakami et al. (2013); Guo et al. (2018); Hao et al. (2021)
	Theabrownin	Pu‐erh tea	225 mg/kg for 10 weeks	Ki67↓; caspase‐3↑; cyclin D1↓; PTEN/Akt/mTOR pathway↓; bacteria producing short‐chain fatty acids, such as Prevotellaceae and Alloprevotella↑; the abundance of Bacteroidaceae and Bacteroides genera↓; anti‐proliferation; pro‐apoptosis; regulation of dysbiosis	Leung et al. (2022)
	BPIS (binding polyphenols in millet bran)	Millet bran	75 and 150 mg/kg, once every 2 days for 10 weeks	COX‐2 and EMR1↓; Ki‐67 and PCNA↓; caspase‐ 3↑; diversity and structure of intestinal microbiota↑	Yang et al. (2020)
	FMBP	Millet bran	100 and 200 mg/kg, once every 3 days for 10 weeks	COX‐2, Ki‐67 and EMR1↓; abundance of PC and PE↓; GPL metabolism↓	Shan et al. (2020)
	Oligonol	Lychee fruit	10, 50, and 100 kg/kg for 3 weeks	NF‐κB and STAT3↓; COX‐2, iNOS and cyclin D1↓; lipid peroxidation and 4‐hydroxy‐2‐nonenal↓; anti‐inflammation; antioxidant; oxidative stress‐induced apoptosis↓	Yum et al. (2013)
	Oleuropein	*Olea europaea*	50 and 100 kg/kg for 8 weeks	IL‐6, IFN‐γ, TNF‐α and IL‐17A↓; COX‐2, Bax and PCNA↓; NF‐κB, Wnt/β‐catenin protein, PI3K/Akt and STAT3 pathway↓; CD4^+^Rorγt^+^IL‐17^+^IFN‐γ^+^ T cell↓; IL‐17A and IFN‐γ↓; anti‐inflammation; anti‐proliferation; pro‐apoptosis and regulation of immunity	Giner et al. (2016)
	Procyanidin B2	Grape seed	10, 20, 30, 50 and 100 mg/kg for 37 days	Colon crypt regeneration	Zhu et al. (2021)

	Resveratrol	Bushy Knotweed	3000 ppm for 8 weeks	Neutrophils infiltratiion in tissue↓; TNF‐alpha and INF‐γ in CD3^+^T cells↑; p53 and phosphorylated serine 15 of p53↓; anti‐ inflammation; regulation of immuntiy	Cui et al. (2010)
	Rosmarinic acid	*Rosmarinus officinalis* L., *Mentha* spp., *Origanum vulgare* L., and *Thymus vulgaris*	30 mg/kg/day for 7 days	TLR4‐mediated NF‐κB and STAT3 activation↓	Jin et al. (2021)
Walnut phenolic extract	Walnut	20 mg/kg/day for 2 weeks	IKK activity and β‐ catenin signaling↓	Koh et al. (2019)
Flavonoid	Ergosterol peroxide	Chaga mushroom	15 mg/kg, twice a day for 14 or 8 weeks	Antiproliferative and pro‐apoptotic activities	Kang et al. (2015)
	Selenoneine	Tuna	0.05% of diet for 48 days	Accumulation of MDSCs↓; IFN‐γ↓	Masuda et al. (2018)
	Fisetin	Strawberries, apples, persimmons, kiwis, mangoes, cucumbers, and onions	20 mg/kg/day for 62 days	Restored antioxidant enzymes; vitamins C and E↓; bax and caspase‐3↑; bcl‐2↓; antioxidant and pro‐apoptotic activities	Kunchari Kalaimathi and Sudhandiran (2016)
	Genistein	Soybeans	5, 15 and 45 mg/kg for 10 days	XO↓; uric acid and TNF‐α↓; GSH↑	Li and Li (2022)
	Genistein‐27	Soybeans	45 mg/kg for 12 weeks	p65‐CDX2‐β‐catenin axis of β‐catenin target genes↓	Du et al. (2016)
	Myricetin	Berries and vegetables	40, 100 mg/kg for 2 weeks	IL‐1β, IL‐6, TNF‐α and COX‐2↓	Zhang, Su, et al. (2018)
	Derivative of myricetin, M10	Berries and vegetables	50 mg/kg/day and 100 mg/kg/day for 12 weeks	Infiltration of MDSCs and pro‐inflammatory mediators↓; CD8^+^T and CD4^+^T lymphocytes↑; ER stress↓	Wang et al. (2018)
	Tricin	Rice and wheat	50 and 150 ppm for 17 weeks	Proliferation index↓; TNF‐α↓	Tanaka et al. (2019)
	Vitexin	Pearl millet, hawthorn, pigeon pea, mung bean, mosses, Passiflora, bamboo, mimosa, wheat leaves, and chaste tree or chasteberry	40 and 80 mg/kg/day for 7 days	Serum NO↑; NO content and M1 macrophage polarization in the colon↑(92); VDR protein↑. CD163↑(26)	Chen et al. (2021, 2024)
	Parthenolide	*Tanacetum parthenium*	2 and 4 mg/kg for 12 weeks	NF‐κB activation↓; Bcl‐2 and Bcl‐extra large↓	Kim, Liu, et al. (2015)
Terphere	Ginsenoside Rb1	*Panax ginseng*	20 mg/kg every 2 days for 18 weeks	TNF‐α, IL‐6, IL‐17A, IL‐33, IL‐1β and IL‐22↓; IL‐10↑; restored the gut dysbiosis	Wang et al. (2023)
	Ginsenoside C‐K	*Panax ginseng*	30 and 60 kg/kg for 13 weeks	Modulating gut microbiota (partially through upregulation of Akkermansia *muciniphila* .)	Shao et al. (2022)
	Ginsenoside derivative, Rh2E2	American ginseng	20, 40, and 100 mg/kg for 3 weeks	ATP production↓; S phase cell cycle arrest	Wong et al. (2016)
	Ziyuglycoside II	*Sanguisorba officinalis* L. roots	0.5 and 1 mg/kg, 3 times per weeks for 64 days	Inhibiting inflammation and inducing apoptosis	Cheon and Kim (2019)
	Apple polysaccharide	Apple	1.25%, 2.5% and 5% of diets for 13 weeks (MAP); 5% of diet for 13 weeks (AP); 2.5%, 5% and 10% of diets for 12 weeks (MAP)	M1 macrophages polarization through modulation of the TLR‐4/NF‐ΚB pathway↑; gal‐3 expression↑	Sun et al. (2018); Sun et al. (2020); Li et al. (2012)
Polysaccharide	APE (polysaccharides extracted from okra flowers)	Okra flower	150 and 300 mg/kg for 17 weeks	Intestinal dysbiosis↓; modulation of Nrf2/IL‐6, MAPKs, PI3K/AKT and Wnt/β‐catenin signaling pathways	Deng et al. (2023)
	Procyanidin B2	*Piper longum* L	10, 20, 30, 50, 100 mg/kg for 30 days	COX‐2 and IL‐6↓; β‐catenin and Snail↓	Huang et al. (2020)
Alkaloids	Neferine	Lotus root	2.5 and 5 mg/kg, 6 consecutive days for 81 days	COX‐2, iNOS, phosphorylated p65 and phosphorylated STAT3↓	Zhou et al. (2022)
	Barley leaf	Barley	2.5% and 5% of diets for 10 weeks	Mucosal barrier function↑; Ki67↓; β‐catenin signaling pathway‐related genes↑; enrichment of Bifidobacterium↑; improve gut dysbiosis	Li et al. (2021)
	Defatted Rice Bran	Rice	3 and 6 g/kg for 13 weeeks	Improve gut dysbiosis	Tajasuwan et al. (2023)
Others	Cocoa	Cocoa	5% and 10% of diets for 8 weeks	Antioxidant and anti‐inflammatory ability	Pandurangan et al. (2015)
	Dietary glutamine	Beans, nuts and vegetables	8.5 g/kg/day for 10 weeks	NF‐κB activity↑; expression of inflammatory proteins↓; DEPTOR↑; phosphorylated mTOR, phosphorylated STAT3, Akt and S6↓; LC3‐II↑; autophagy↑	Tian et al. (2016)
	Phycocyanin	*Spirulina platensis*	50 and 100 mg/kg for 12 weeks	Firmicutes, Bacteroidetes, Proteobacteria, and Verrucomicrobia↓; gene expression involved in intestinal barrier function↑; IL‐17 signaling pathway↓	Pan et al. (2022)

### 4.1 Phenols

Previous literature has reported that various dietary phenolic compounds play an important role in the treatment and/or prevention of AOM/DSS‐induced inflammatory‐related colon cancer in rats, for example, 6‐gingerol (6‐G), carnosol (CS), goniothalamin (GTN), carvacrol (CARV), curcumin (CUR), theabrownin (TB), bound polyphenol of the inner shell (BPIS), peroxidase from foxtail millet bran (FMBP), oligonol (OLG), oleuropein (OLE), procyanidin B2 (PB2), resveratrol (RE), rosmarinic acid (RA), and walnut phenolic extract (WPE). Furthermore, their effects and mechanisms are illustrated in Table 1 and Figure 5A.

6‐Gingerol (6‐G), a key aromatic polyphenolic compound found in ginger, may have therapeutic potential in preventing AOM‐induced colon cancer by influencing antioxidant activity and inflammation (Aloliqi 2022). Ebele et al. demonstrated that in a mouse model of ulcerative colitis, 6‐G has anti‐inflammatory and antioxidative effects by modulating the levels of different cytokines and antioxidant enzymes. Furthermore, in the AOM/DSS‐induced CRC (CAC) model, 6‐G treatment significantly lowers the incidence of adenocarcinoma by reducing the expression of β‐catenin and cyclin D1 (Farombi et al. 2020).

CS is a potent antioxidant phenolic compound in mustard seed oil (Shrestha et al. 2012) and crude canola (rapeseed) oil (Fang et al. 2013). A diet that includes CS significantly reduces the symptoms and the pathological progression of DSS‐induced colitis. Moreover, CS notably lowers the production of inflammatory cytokines like IL‐12 and TNF‐α. In the AOM/DSS‐induced CAC model, mice given CS show a lower rate of cancer occurrence and tumor burden. Additionally, inflammatory cytokines such as COX‐2, iNOS, and TNF‐α, as well as molecules involved in oxidative response like heme oxygenase‐1, are reduced. These findings suggest that CS may have chemopreventive effects during the initiation and promotion phases of carcinogenesis (Fang et al. 2013).

GTN is a styryl lactone found in plants of the genus *Goniothalamus*, Annonaceae, such as cherimoya. Earlier research has shown that GTN decreases the number, burden, and size of tumors in an AOM/DSS‐induced CAC model. Furthermore, GTN lowers the levels of IL‐6, IL‐17, and TNF‐α in tumor tissue and prevents the activation of stromal immune cells and the nuclear translocation of NF‐κB (Vendramini‐Costa et al. 2017).

CARV is a phenolic monoterpene extracted from *Oreganum vulgare* essential oil. In a rat CAC model induced by DMH/DSS, CARV treatment boosts the activation of natural antioxidant systems, reduces the expression of inflammatory mediators, improves the antioxidant status in intestinal mucosal inflammation, and facilitates the recovery of tissue pathology (Arigesavan and Sudhandiran 2015).

Curcumin (CUR) is a polyphenol found in spices often included in diets. It comes from the perennial plant *Curcuma longa* Linn., which is part of the ginger family. Because it can interact with several molecular targets involved in different stages of cancer development, CUR is being researched as a potential new cancer treatment. In fact, preclinical studies have demonstrated that CUR has positive effects on a variety of cancers and can effectively block carcinogenesis at various points in cancer progression (Thangapazham et al. 2008). Moreover, CUR can induce apoptosis in cancer cells by interfering with multiple signaling pathways (Villegas et al. 2008). Interestingly, increasing preclinical evidence suggests that CUR is a promising candidate for addressing CRC progression (Villegas et al. 2011; Murakami et al. 2013; Guo et al. 2018; Hao et al. 2021; Seiwert et al. 2021). Earlier research has demonstrated that animals treated with DSS develop smaller tumor lesions, with β‐catenin migrating into the cytoplasm and/or nucleus within the tumor tissues. However, following dietary supplementation with CUR, this translocation and its intensity are notably reduced, suggesting that CUR plays a protective or preventive role in the progression of CAC (Villegas et al. 2011). Another study revealed that in an AOM/DSS‐induced CAC mouse model, both turmeric extract (TUR) and CUR used separately significantly inhibit the multiplicity of adenomas. Interestingly, the combined use of CUR and TUR eliminates tumor formation (Murakami et al. 2013). Epigenetic research in mice has demonstrated that CUR treatment can reverse the reduced DNA CpG methylation of the TNF gene caused by AOM/DSS and lower the DNA methylation levels of genes associated with inflammation (Guo et al. 2018). In addition, the anticancer effect of CUR can be achieved by inhibiting the expression of proinflammatory cytokines and reducing the expression of Axin2 in the Wnt/β‐catenin protein signaling pathway (Hao et al. 2021). Further research has shown that the administration of nanosized micelle‐formulated curcumin (mCur) to drinking water significantly reduces AOM/DSS‐induced colitis and colon cancer formation (Seiwert et al. 2021).

TB is a polyphenolic compound with oxidative properties, extracted from Pu‐erh tea, a variety of black tea. Studies indicate that TB significantly lowers the total number of tumors caused by AOM/DSS and improves the depth of the crypts and fibrosis in the colon. TB treatment reduces the levels of the proliferation marker Ki‐67, whereas boosting the expression of caspase‐3. Additionally, TB notably suppresses the PTEN/Akt/mTOR signaling pathway and its downstream target protein, cyclin D1, which may help inhibit cell proliferation and enhance apoptosis. Furthermore, TB increases the presence of bacteria that produce short‐chain fatty acids, such as Prevotellaceae and Alloprevotella, whereas reducing the abundance of Bacteroidaceae and Bacteroides, which are linked to colon cancer (Leung et al. 2022).

BPIS is a millet bran extract that is mainly composed of 12 phenolic acids. BPIS and its main active ingredients (caffeic acid and p‐coumaric acid) exhibit significant anticancer activity in vivo and in vitro (Shi et al. 2017). Additionally, BPIS slows tumor growth in the AOM/DSS‐induced CAC model by decreasing the levels of COX‐2, EMR1, and PCNA, whereas activating caspase‐3. This protein is also involved in restoring the diversity and structure of the gut microbiota and regulating several functional genes (Yang et al. 2020).

Abnormal glycerophospholipid metabolism is a typical metabolic feature of cancer, playing a role in tumor progression, especially in the metabolism of phosphatidylcholine (PC) and phosphatidylethanolamine (PE). Notably, Shan et al. found that treatment with FMBP effectively prevents the increased expression of early CRC markers (COX‐2, Ki‐67, and EMR1) in mice with CAC. Additionally, FMBP treatment significantly reduces the levels of PC and PE involved in glycerophospholipid (GPL) metabolism. Furthermore, FMBP lowers the expression of key metabolic enzymes involved in the metabolism of PE and PC, thereby inhibiting CRC growth by disrupting GPL metabolism and causing ATP depletion (Shan et al. 2020).

OLG, a polyphenol from lychee fruit, significantly blocks the activation of NF‐κB and STAT 3, and reduces the expression of COX‐2, iNOS, and cyclin D1 in the mouse colon. Additionally, OLG protects mice from colitis induced by DSS and prevents the formation of adenomas in the colon induced by AOM/DSS. OLG also lowers lipid peroxidation (malondialdehyde) and protein oxidation (4‐hydroxy‐2‐nonenal), helping to prevent oxidative stress‐induced cell death in colonic epithelial cells (Yum et al. 2013).

OLE, a key phenolic secoiridoid, is found in the leaves and unripe fruits of * *Olea europaea* * (olive tree). According to Elisa et al., OLE helps protect against AOM/DSS‐induced CAC by improving clinical symptoms, lowering disease activity index scores, and inhibiting tumor growth and diversity. Further studies show that OLE treatment reduces intestinal levels of IL‐6, IFN‐γ, TNF‐α, and IL‐17A, and lowers the expression of COX‐2, Bax, and PCNA. Notably, OLE treatment significantly downregulates cancer‐related pathways, including NF‐κB, Wnt/β‐catenin, PI3K/Akt, and STAT3. Additionally, in the DSS‐induced acute colitis model, OLE suppresses Th17 responses by decreasing the CD4+ Rorγt+ IL‐17+ IFN‐γ + T‐cell subset in the submucosal layer of the intestines, along with lower expression levels of IL‐17A and IFN‐γ (Giner et al. 2016).

In addition, procyanidin B2 (PB2) extracted from grape seeds (Zhu et al. 2021) RE extracted from the Bushy Knotweed plant (Cui et al. 2010), RA extracted from some culinary herbs of the Labiatae family, such as *Rosmarinus officinalis* L., *Mentha* spp., *Origanum vulgare* L., and *Thymus vulgaris* (Jin et al. 2021), and WPE (Koh et al. 2019) were shown to significantly reduce tumor development in a murine model of CAC induced by AOM/DSS. PB2‐mediated alleviation of CAC may be associated with the ability of PB2 to promote colon crypt regeneration (Zhu et al. 2021). RE greatly enhances inflammation scores, lowers the proportion of neutrophils in mesenteric lymph nodes and mucosal sublayers, and influences the expression of TNF‐α and IFN‐γ in CD3+ T cells. It also decreases the levels of inflammation markers and inflammatory stress (p53 and phosphorylated serine 15 of p53) (Cui et al. 2010). RA suppresses CAC by propitiating TLR4‐mediated NF‐*κ*B and STAT3 activation pleiotropically (Jin et al. 2021). WPE attenuates tumor growth by inhibiting IKK activity and β‐catenin signaling independent of the induction of apoptosis in an AOM/DSS‐induced CAC model (Koh et al. 2019).

### 4.2 Flavonoids

In the AOM/DSS‐induced rat inflammatory‐associated colon cancer model, dietary flavonoids such as kaempferol (Kae), ergosterol peroxide (EP), selenoneine (SeN), fisetin (FS), genistein (GEN), myricetin (MYR), TRI and vitexin (Vit) exert specific therapeutic effects on CAC through different mechanisms, as illustrated in Table 1 and Figure 5B.

Kaempferol (Kae) is a flavonoid mainly found in the Zingiberaceae family of plants, which includes several spices native to India. Shirley et al. found that Kae treatment significantly reduces the tumor burden caused by AOM through alterations in miRNA expression, regulation of sphingolipid metabolism, and modulation of the ErbB signaling pathway (James et al. 2021).

EP, extracted from Chaga mushrooms (*Inonotus obliquus*), has been found to inhibit tumor growth induced by AOM/DSS and reduce the number of Ki67‐positive colon epithelial cells while increasing the number of TUNEL‐stained cells, which is related to its antiproliferative and proapoptotic activities (Kang et al. 2015).

An analog of ergothioneine, selenoneine (SeN), which contains selenium (Se), is the primary organic selenium form found in tuna blood, muscles, and other tissues (Yamashita and Yamashita 2010). Oral administration of SeN notably reduces tumor incidence and suppresses the accumulation of myeloid‐derived suppressor cells (MDSCs) while preventing the decrease in IFN‐γ production during AOM/DSS‐induced carcinogenesis (Masuda et al. 2018).

FS is a flavonoid in various fruits and vegetables, such as strawberries, apples, persimmons, kiwis, mangoes, cucumbers, and onions (Kim, Kim, et al. 2015). Kunchari et al. found that in a mouse model of AOM/DSS‐induced CAC, FS treatment reduced the levels of key tumor markers like 5′‐nucleotidase and the oxidative stress marker γ‐glutamyl transferase. FS also helped restore antioxidant enzymes and the breakdown of vitamins C and E. FS administration notably increased the protein expression of bax and caspase‐3, whereas reducing the expression of bcl‐2. These findings suggest that FS offers both preventive and therapeutic benefits for CAC through its antioxidant and proapoptotic effects (Kunchari Kalaimathi and Sudhandiran 2016).

GEN is a natural isoflavone found in soybeans. Recent research has revealed that xanthine oxidase (XO), an enzyme involved in the production of uric acid and reactive oxygen species during nucleic acid metabolism, can contribute to tumor growth. In the AOM/DSS‐induced CRC (CAC) model, GEN significantly reduces the number of tumors, decreases XO expression and activity, lowers uric acid and TNF‐α levels in the colon tissue, and boosts the levels of reduced glutathione (GSH) in the colon. These findings suggest that GEN helps delay colon tumor progression by inhibiting XO activity (Li and Li 2022). Further research revealed that GEN‐27, a derivative of isoflavone, reduces mortality, tumor number, and tumor volume in mice. The mechanism of the preventive effect of GEN‐27 on CAC may involve the inhibition of the p65‐CDX2‐β‐catenin axis of β‐catenin target genes (Du et al. 2016).

MYR is a flavonoid found in natural sources like berries and vegetables. Studies have demonstrated that MYR helps alleviate colitis symptoms and notably reduces both the number and size of tumors in a mouse model. Additional analysis showed that MYR lowers the levels of proinflammatory cytokines, including IL‐1β, IL‐6, TNF‐α, and COX‐2, in colon adenomas (Zhang, Su, et al. 2018). Additional studies showed that orally administered M10, a new derivative of MYR, helps prevent ulcerative colitis (UC) and colorectal tumors in mice. The chemopreventive effect works by reducing the infiltration of MDSCs and proinflammatory substances in the colon tissues and bloodstream. It also boosts the levels of CD8+ and CD4+ T lymphocytes and lowers macrophage activity, cell proliferation, and autophagy, primarily by inhibiting strong ER stress in colorectal epithelial cells (Wang et al. 2018).

TRI, found in rice and wheat, greatly inhibits the development of AOM/DSS‐induced CRC. It also significantly decreases the proliferation rate, mitotic count, and cell cycle duration of adenocarcinoma cells, whereas notably reducing the expression of TNF‐α in normal‐appearing crypts (Tanaka et al. 2019).

Vitexin (Vit), a c‐glycosylated flavone, is found in pearl millet, hawthorn, pigeon pea, mung bean, moss, Passiflora, bamboo, mimosa, wheat leaves, and chaste tree or chasteberry in seeds, fruits, flowers, leaves, and roots, etc. (He et al. 2016). In a mouse model of CAC induced by AOM/DSS, oral administration of Vitamin (Vit) notably improves the symptoms of chronic colitis, decreases liver damage in the colon, lowers tumor occurrence, and reduces the overall tumor load. Further investigation into the mechanisms showed that Vitamin (Vit) influences the levels of proinflammatory substances around adenomas, boosts serum nitric oxide (NO) levels and NO content in the colon, and promotes M1 macrophage polarization in the colons of CAC mice (Chen et al. 2021). Recently, Chen et al. found that vitexin has been shown to directly target VDR, binding to the VDR‐LBD domain, thereby promoting its translocation to the cell nucleus. This process then regulates the transcription of PBLD and affects macrophage polarization. These results indicate that the direct targeting of VDR by vitexin alters macrophage polarization and thus inhibits the progression of colitis to CRC (Chen et al. 2024).

### 4.3 Terpenes

As shown in Table 1 and Figure 5C, dietary terpenoid compounds, including parthenolide (PT), American ginseng extract contains ginsenosides (Rg1, GC‐K, and Rh2E2), and ziyuglycoside II significantly inhibited AOM/DSS‐induced CAC in a rat model by various molecular mechanisms.

PT, which is a natural sesquiterpene lactone originally purified from *Tanacetum parthenium* , inhibits carcinogenesis by inhibiting NF‐κB activation, reducing the expression of the antiapoptotic proteins Bcl‐2 and Bcl‐XL (Kim, Liu, et al. 2015).

American ginseng extract contains ginsenosides (Rg1, Re, Rb1, Rc, Rb2, and Rd) and additional ginsenosides composed of F11, Ro, isomers of Rd, and traces of malonyl ginsenosides and ginseng root‐derived polysaccharides/oligosaccharides and proteins (Jin et al. 2008). Ginsenoside Rb1, one of the major active components of *Panax ginseng* (Chinese herb) (Wang et al. 2023), and ginsenoside C‐K, which is produced from ginsenoside Rb1 (Shao et al. 2022), have been reported to significantly attenuate AOM/DSS‐induced colon carcinogenesis. Further research revealed that ginsenoside Rb1 may inhibit tumor growth by alleviating chronic inflammation and restoring the intestinal microenvironment (Wang et al. 2023). Additionally, GC‐K can suppress tumor growth by modulating the gut microbiota (partially through the upregulation of *A. muciniphila* ). In addition, the ginsenoside derivative Rh2E2 specifically inhibits ATP production in cancer cells by downregulating metabolic enzymes involved in glycolysis, fatty acid β‐oxidation, and the tricarboxylic acid cycle, leading to specific cytotoxic effects on cancer cells and S phase cell cycle arrest (Wong et al. 2016). In addition, Ziyuglycoside II, a triterpenoid saponin found in *Sanguisorba officinalis* L. roots, significantly reduces AOM/DSS‐induced CAC by inhibiting inflammation and inducing apoptosis (Cheon and Kim 2019).

### 4.4 Polysaccharides

The polysaccharides from apple and okra flowers exerted a suppressive effect on AOM/DSS‐induced CAC in the rat model by various molecular mechanisms, as shown in Table 1 and Figure 5D.

Growing experimental evidence suggests that apple polysaccharide (AP) can prevent the onset and progression of CAC through various mechanisms. Research by Sun et al. proposed that AP inhibits CAC by influencing pathological changes in IECs and cell signaling pathways (Sun et al. 2018). Additional studies found that AP helps protect against CAC by influencing macrophage polarization, specifically promoting the shift of macrophages to the M1 phenotype via the TLR‐4/NF‐κB pathway (Sun et al. 2020). Another study indicated that AP derived from apple pomace and pulp exhibits anti‐CAC properties, potentially involving mechanisms that affect B‐galactoside‐binding lectin (gal‐3) expression and function (Li et al. 2012). Furthermore, APE (polysaccharides extracted from okra flowers) effectively reduces colitis and the formation of CRC (CAC), whereas also improving gut microbiome imbalance. Furthermore, the anticancer effects of APE may be linked to the modulation of various signaling pathways, including Nrf2/IL‐6, MAPK, PI3K/AKT, and Wnt/β‐catenin, in the AOM/DSS‐induced CAC mouse model (Deng et al. 2023).

### 4.5 Alkaloids

The active alkaloid compounds, procyanidin B2 (PB2) and Neferine (Nef), significantly inhibited the occurrence and development of CAC through different mechanisms (as shown in Table 1 and Figure 5E).

Procyanidin B2 (PB2) is an active alkaloid isolated from *Piper longum* L (Tripathi and Biswal 2020). Huang et al. reported that PB2 can inhibit colitis and reduce the number of large colorectal tumors in mice by downregulating COX‐2 and IL‐6, as well as β‐catenin and Snail (Huang et al. 2020).

Neferine (Nef), a natural dibenzyl isoquinoline alkaloid, significantly suppresses DSS‐induced experimental ulcerative colitis in mice (Wu et al. 2018; Min et al. 2021). Recent studies have demonstrated that oral Nef substantially decreases both the number and size of tumors. Additionally, Nef reduces the infiltration of inflammatory cells and epithelial hyperplasia, whereas also lowering the levels of proinflammatory cytokines in colon tissues. Furthermore, Nef significantly decreases the protein expression of COX‐2, iNOS, phosphorylated p65, and phosphorylated STAT3 in both tumor and nontumor tissues. Molecular docking analyses showed interactions between Nef and NF‐κB p65, as well as between Nef and STAT3. In conclusion, these findings suggest that Nef may inhibit the carcinogenic process in CAC by modulating NF‐κB and STAT3 signaling pathways (Zhou et al. 2022).

### 4.6 Others

As shown in Table 1 and Figure 5F, in addition to the aforementioned dietary phenolic compounds, flavonoids, terpenes, polysaccharides, and bioactive alkaloids that have a significant inhibitory effect on AOM/DSS‐induced CAC in rats, the literature also reports on the therapeutic effects and mechanisms of some functional foods and other categories of dietary compounds on CAC.

Barley leaf (BL) is rich in natural nutrients and is a functional food with various health‐promoting activities (Benedet et al. 2007). Snider et al. found that barley leaf supplements can decrease tumor development and tissue damage. This effect is linked to the inhibition of inflammatory enzymes tied to colitis, improvements in mucosal barrier function, a reduction in elevated cell proliferation markers, increased expression of genes related to the β‐catenin signaling pathway, and prevention of AOM/DSS‐induced gut dysbiosis by boosting the growth of Bifidobacterium (Li et al. 2021).

Cocoa is a rich source of polyphenols, with cocoa beans containing 6%–8% total polyphenols. A 5% cocoa diet most completely reduces tumor size in AOM/DSS‐treated mice, whereas mice on a 10% cocoa diet present a proliferative colon mucosa with reduced development of crypts and buds, which was associated with its antioxidant and anti‐inflammatory ability (Pandurangan et al. 2015).

Tian et al. reported that in a mouse model of AOM/DSS‐induced CAC, glutamine (GLN) exhibits protective effects (Tian et al. 2016, 2013) associated with the inhibition of inflammation and NF‐κB activity, as well as decreased overexpression of inflammatory proteins (Pan et al. 2022). Furthermore, The administration of GLN increases the expression of DEPTOR and decreases the expression of factors in the mTOR signaling pathway, such as phosphorylated mTOR, phosphorylated STAT3, phosphorylated Akt, and phosphorylated S6. Furthermore, oral GLN administration is linked to an increase in LC3‐II expression in AOM/DSS‐treated mice. These findings suggest that the DEPTOR/mTOR signaling pathway could play a key role in GLN's prevention of CAC development. Additionally, the chemopreventive effect of dietary GLN on CAC appears to be partly due to the induction of autophagy (Tian et al. 2016).

Phycocyanin (PC) is a pigment‐protein complex derived from spirulina (Arthrospira platensis). In the AOM/DSS‐induced CAC model, PC helps reduce inflammation and lowers the number of colon tumors. Mechanistic studies have shown that PC inhibits the proliferation of epithelial cells in CAC mice. It also decreases the relative abundance of Firmicutes, Bacteroidetes, Proteobacteria, and Verrucomicrobia at the phylum level. Additionally, PC treatment enhances the expression of genes related to intestinal barrier function. KEGG pathway analysis indicates that PC treatment impacts the IL‐17 signaling pathway (Pan et al. 2022).

Furthermore, defatted rice bran could serve as a prebiotic supplement to regulate gut microbiota dysbiosis, potentially lowering CRC risk (Tajasuwan et al. 2023).

## 5 Conclusion and Future Perspective

The pathogenesis of CAC has not yet been clearly described. The application of the AOM/DSS‐induced CAC model is crucial for elucidating the pathogenic mechanisms of colorectal inflammation‐associated cancer, from inflammatory signaling pathways and antioxidant mechanisms to the impact of microbiota communities and the various changes in gene and protein expression. Therefore, the AOM/DSS‐induced CAC model serves as a robust platform for studying the pathogenesis of inflammatory CRC and developing effective and safe new drugs. Food and food‐derived biologically active compounds have been extensively studied as potential chemopreventive agents for CRC. Many studies have specifically documented the protective effects of dietary compounds on colitis and CAC in clinically relevant disease models. This article provides the latest information on food‐derived biologically active compounds for the prevention and treatment of AOM/DSS‐induced CAC, guiding the selection of dietary compounds for treating inflammation‐associated CRC and offering new insights for the development of novel CAC therapeutic drugs.

At present, although many food‐based compounds have demonstrated encouraging results in preclinical studies on AOM/DSS‐induced CAC, clinical trials confirming their effectiveness in treating CAC are still lacking. To improve CAC management, well‐structured randomized clinical trials are needed to assess the effectiveness of these compounds and to explore the potential of new molecules. The progress in bioinformatics, network pharmacology, molecular docking, Mendelian genetics, and other fields related to drug development offers a promising path for the continued advancement of safe and effective food‐derived compounds for preventing and treating CAC.

## Author Contributions

**Zhong‐Yang Yu:** supervision, writing – original draft, writing – review and editing, methodology, project administration. **Jin‐Wei Zhao:** conceptualization, funding acquisition, writing – original draft, writing‐review and editing. **Wei‐Yi Zhao:** investigation, writing – original draft, visualization.

## Conflicts of Interest

The authors declare no conflicts of interest.

## Data Availability

The datasets used and analyzed during the current study are available in the Pubmed repository https://pubmed.ncbi.nlm.nih.gov/ or available from the corresponding author upon reasonable request.
